# Treated Colorectal Cancer: What is the Cost to Primary Care?

**DOI:** 10.4137/cmo.s877

**Published:** 2008-12-22

**Authors:** D.A.L. Macafee, J. West, J.H. Scholefield, D.K. Whynes

**Affiliations:** 1 Specialist Registrar in General Surgery, Department of Paediatric Surgery, Royal Victoria Infirmary, Newcastle upon Tyne.; 2 Specialist Registrar in Gastroenterology and Clinical Scientist, Division of Epidemiology and Public Health, University of Nottingham, Queen’s Medical Centre, Nottingham.; 3 Professor of Surgery, Division of Gastrointestinal Surgery, Wolfson Digestive Diseases Centre, Nottingham University Hospitals, Queen’s Medical Centre, Nottingham NG7 2UH.; 4 Professor of Health Economics, School of Economics, University of Nottingham.

**Keywords:** colorectal cancer, cost, general practice, Dukes stage, stoma

## Abstract

**Background:**

Colorectal cancer is the second commonest cause of cancer death and the cost to primary care has not been estimated.

**Aim:**

To determine the direct primary care costs of colorectal cancer care.

**Design:**

Retrospective case note review.

**Setting:**

Nottingham, United Kingdom.

**Methods:**

We identified people with colorectal cancer between 1995 and 1998, from computerised pathology records. Colorectal cancer related resources consumed in primary care, from hospital discharge to death, were identified from retrospective notes review. Outcome measures were costs incurred by the General Practitioner (GP) and the total cost to primary care. We used multiple linear regression to identify predictors of cost.

**Results:**

Of 416 people identified from pathology records, the median age at primary operation of the 135 (33%) people we selected was 74.2 (IQR 14.4) years, 75 (56%) were male. The median GP cost was: Dukes A £61.0 (IQR 516.2) and Dukes D £936.2 (1196.2) p < 0.01. The geometric mean ratio found Dukes D cancers to be 10 times as costly as Dukes A. The median total cost was: Dukes A £1038.3 (IQR 5090.6) and Dukes D £1815.2 (2092.5) p = 0.06. Using multivariate analysis, Dukes stage was the most important predictor of GP costs. For total costs, the presence of a permanent stoma was the most predictive variable, followed by adjuvant therapy and advanced Dukes stage (Dukes C and D).

**Conclusions:**

Contrary to hospital based care costs, late stage disease (Dukes D) costs substantially more to general practice than any other stage. Stoma care products are the most costly prescribable item. Costs savings may be realised in primary care by screening detection of early stage colorectal cancers.

## Introduction

Colorectal cancer remains the second commonest cause of cancer death in the U.K. and consumes significant resources within both primary and secondary care.^1^ Previous studies have looked at the costs of hospital based care, finding the costs of very early and very late stage cancers to be significantly lower than those of treating cancers in the intermediate stages.^2^ However, there is scarce information on the resources consumed by treated colorectal cancer patients following hospital discharge. The resource consumption of this common cancer may be considerable and costly so our aim, in this retrospective study, was to establish the cost that treated colorectal cancer incurred on primary care.

## Methods

### Study population

We identified people with histologically proven colorectal cancer, treated at one hospital between 1995 and 1998, from computerised pathology records. People identified as deceased, had their notes reviewed by a single investigator (DM) at the local health authority. A small group of people still alive (n = 8) and registered with two GP practices, also had their notes reviewed. The study time period was from hospital discharge following excision of the primary cancer until death or the study end date (01/01/2003). We excluded those patients who died in the early post-operative period (30 days) without being discharged home, as they had consumed no community resources. Ethical approval was obtained for the study (LREC Q1110208).

### Costs

Our main outcomes were costs incurred by the General Practitioner (GP) and the total cost to primary care. We included resources consumed in primary care and related to colorectal cancer and excluded costs due to benign gastrointestinal symptoms (e.g. constipation). GP costs included all GP related activity (e.g. home visits) or prescribing. Total cost to primary care, included all identifiable costs related to colorectal cancer care (e.g. district nurse, stoma care products) in addition to the GP costs. The cost of training a GP was considered when costing their time (qualification costs) and a 5% discount rate was chosen for costs in the main analysis. We used standard sources to calculate costs,^3^–^7^ which were then extrapolated to 2002 prices, using the Gross Domestic Product deflator.^8^ Drugs prescribed by GP’s were costed for a one month supply. An estimated cost (£1500) of yearly stoma care products was included in the calculation of total cost, based on each person using 2 stoma bags per day at a unit cost of £2, plus other occasional consumables.

### Statistical analysis

Initially we described the characteristics and cost data by Dukes stage of disease using median values, interquartile ranges and non-parametric tests where appropriate, as the cost data was non-normal. We used a non-parametric test for trend^9^ to examine trends across Dukes stage. We excluded 5 subjects from the regression analysis who had incurred no primary care cost and so were considered outliers. Following log_e_ transformation of costs, multiple linear regression (backward variable elimination technique) was used to estimate the best predictors of primary care costs. Sensitivity analysis was performed across the three different GP costs structures, cost of stoma care (between £1000 and £2000) and through a range of discounting rates (0%–10%). Analyses were performed in Stata 7.0 (Stata Corporation, Texas).

## Results

Of 416 people identified from pathology records, 127 were deceased. Within two GP practices, 8 people still alive had their notes reviewed. Overall, 22 (5%) subjects were excluded due to death in the peri-operative period. The median age at primary operation, of the 135 (33%) people included, was 74.2 (IQR 14.4) years, 75 (56%) were male and 112 (83%) of primary operations were considered curative. The median time from operation to death or end date was 2.1 years (IQR 2.4); those with a Dukes A cancer surviving longest—3.1 years (IQR 2.1); Dukes D surviving least long −0.8 years (IQR 0.9). The proportion of people dying from colorectal cancer increased across stage −11% in Dukes A and 95% in Dukes D (Table 1).

Table 2 presents the GP costs and total costs by various demographic, disease and treatment characteristics. There were large differences in GP cost across Dukes stage, which showed a trend of rising costs with more advanced disease (Dukes D versus Dukes A was £936.2 vs. £61.0; p ≤ 0.01). The pattern was similar, although not statistically significant, for total cost (Dukes D versus Dukes A was £1815.2 vs. £1038.3; p = 0.06). People aged 75 years or over at operation incurred less costs than those under 75: £351.8 vs. £669.6 (p = 0.01); total costs were £926.0 vs. £1608.9 (p < 0.01). Having a permanent stoma, although not statistically significant in the cost of GP care, had a large effect on total cost. Cost of managing a permanent stoma versus no stoma was £3766.3 vs. £664.9 (p < 0.05). Sensitivity analysis was performed, varying the cost structure, stoma care costs and the discounting rate but this made no difference to the overall conclusions.

Table 3 illustrates the effect of various characteristics on cost, when selected using backward elimination regression analysis. Dukes stage and adjuvant therapy were the only variables that remained in the model for GP costs, which rose incrementally with advancing stage. Once adjusted for adjuvant therapy, the ratio of the geometric mean GP cost of people with Dukes D cancer compared to Dukes A was 10:1 (95% CI 4.0–25.0). When we analysed total cost, Dukes stage appeared to be less important in contrast to having a permanent stoma where costs versus no stoma were 7-fold more expensive (geometric mean ratio 7.4 (95% CI 5.0–11.0)). The total cost of a Dukes D cancer was twice that of Dukes A (geometric mean ratio 2.2 (95% CI 1.2–3.8)).

## Discussion

### Findings

We found that Dukes stage was the most important determinant of GP costs in those treated for colorectal cancer, with Dukes D cancers costing approximately ten times as much as Dukes A cancers. The trend of rising cost with advancing stage seen in this study, has not been observed in hospital based care where very early and very late stage conferred least costs. In contrast, Dukes stage appeared less important than the presence of a permanent stoma when calculating total cost to primary care, probably because of the expense and consistent requirement of stoma products (£1500–£2000 per annum). However, on average, a Dukes D cancer still cost £777 more than a Dukes A cancer.

### Strengths and limitations

Reviewing the notes of deceased patients limited our study population to a more advanced stage of disease with a reduced life expectancy but ensured an accurate end point for costs. The eight alive patients included were the result of the pilot period of this study and were therefore part of the study design. When excluded, the high proportion of permanent stomas in the small group of Dukes A cancers (50%) and their long survival period (median 3.8 years) caused the total costs of Dukes A to be higher than any other—£3043.8 (IQR 5628.1). Notwithstanding this, the cost of Dukes A to the GP remained minimal, total costs for Dukes B and C remained over £670 cheaper than Dukes D. When costs were annualised, the cost per life year showed an even greater trend of rising cost with advancing stage. As most recurrences occur within two years study time period provided up to 8 years of follow up^10^ we would expect that any recurrent disease had been detected and therefore costed.

Although irresectable (often advanced stage) disease is one indication for a permanent stoma, the decision is also often dependent on the site of the lesion. Regular stoma care products are required for those with permanent stomas, yet the results of GP costs indicate that this cost is rarely documented. Estimated costs were required due to the lack of documentary evidence and minimal economic data in the medical literature. We used a figure of £1500 which assumes the use of two bags per day plus additional consumables, an estimate deemed reasonable by an experienced stoma care nurse (personal communication Ms. J. Watts). When we varied the annual stoma cost by £1000 it made no difference to the overall conclusions of our study.

Identifying actual resource use is a more robust and accurate method of economic analysis than economic modelling but it is time-consuming and expensive. Economic modelling must estimate resource consumption but in doing so, makes assumptions of use and costs. Our study reduced the number of such assumptions that had to be made.

Hospital based follow up during the 1990’s would probably be considered less protocol driven in the U.K. than it is today. CEA measurements whether in hospital or primary care were not routinely used (and in certain institutions would remain debatable). USS surveillance for liver metastases has been superceded by CT Chest, Abdomen and Pelvis surveillance, although the frequency of tests (including colonoscopy) has perhaps reduced. Generally, in Nottingham at the time, follow up was hospital based under the original consultant. It was not possible to review the travel expenses aspect and a future study of the patient costs incurred would be useful.

Who should perform the follow up and how intensively remain hotly debated issues but studies have shown that primary care can perform this role although the research is perhaps slightly dated.^11^ Renehan et al. concluded from a meta-analysis of intensive hospital based follow up that “based on the available data and current costs, intensive follow up after curative resection for colorectal cancer is economically justified and should be normal practice.”^12^ As with any meta-analysis there must be a degree of caution in potentially costly decisions. A Norwegian trial of systematic follow up found that follow-up program did not influence cancer-specific survival, that overall compliance with the surveillance program was 66% and the total program cost was 20,530 euro (U.S. 25,289 dollars) for one surviving patient after surgery for recurrence.^13^ In a study by our unit, a simulated intensive follow up programme would detect considerably more resectable recurrences but the financial cost and resource requirements would be considerable. Resource limitations, the introduction of screening and continuing NHS deficits may mean that the most intensive follow up will need to be tailored to those with the highest likelihood of recurrent CRC.^14^

We have only dealt with costs related to colorectal cancer but we may have underestimated the palliative care costs due to the limited documentation of District and other Nurse activity in GP records. In palliative care settings, 71.6% of patients require District nurse input with only 10% utilising GP’s and home helps.^3^ In addition, having taken a health service perspective rather than a patient (or societal) viewpoint we have had to ignore costs that are subsequently transferred from hospital care to the patient and his/her household.^15^ Mean patient and carer costs of between £404 to £914 have been found in those undergoing palliative chemotherapy, whilst the mean total primary care costs in the same study were only £114–£152.^16^ If such costs had been included, the trend of rising total costs with advancing stage might have been more striking.

### Implications

Subjects successfully operated for an early stage colorectal cancer without the creation of a stoma, had minimal ongoing primary care costs. Advanced stage disease or a permanent stoma required substantial resource allocation and despite overlooking patient and carer costs, advanced stage disease cost primary care £900 per year more than earlier stage cancers. This is a considerable cost and should be considered when apportioning resources.

Ours is the first study that has looked at the primary care costs for treated colorectal cancer. From a hospital perspective, it is often considered that Dukes D confers minimal cost due to reduced life expectancy but this study has shown this to be an unreliable assumption. While hospital costs may be low, primary care costs are generally higher. Reducing the number of permanent stomas may be helped by modern surgical techniques and stapling devices, which enable very low rectal anastomoses and ileo-anal pouch formations. Identifying earlier stage disease can be achieved by screening, confirmed recently in the national FOBT pilot screening program,^17^ so reducing the costs of advanced disease to primary care.

## Where this Piece Fits?

There are over 34,000 new cases of colorectal cancer in the U.K. per year, costing over £300 million in surgical, oncological and palliative care services. Previous hospital based studies have found Dukes A and D cancers to cost less than intermediate stage cancers but the cost to primary care has not been established and is likely to be considerable. Our study found that GP costs increased with advancing stage. Stoma care products also added significantly to primary care costs but were independent of stage. Costs savings may be realised in primary care by screening detection of early stage colorectal cancers.

## Figures and Tables

**Table 1:** Characteristics of the 135 patients within the study group by Dukes stage.

	**Dukes stage of disease**			
	**A (n = 9)**	**B (n = 34)**	**C (n = 73)**	**D (n = 19)**
Male n (%)	5 (56)	17 (50)	38 (52)	15 (79)
Age at operation Years (IQR)	75.5 (15.6)	77.0 (9.9)	72.9 (14.8)	71.5 (15.8)
Permanent stoma required n (%)	4 (44)	11 (32)	16 (22)	9 (47)
Intensive nursing required n (%)	1 (11)	3 (9)	4 (6)	1 (5)
Adjuvant therapy required n (%)	2 (25)	11 (32)	40 (55)	7 (37)
Deaths from colorectal cancer n (%)	1 (11)	15 (44)	56 (77)	18 (95)

**Table 2:** GP and total costs by various characteristics and Dukes stage.

	Median GP costs only £ (IQR)*	**p value**	Median total cost £ (IQR)*	**p value**	**Median time from operation to death years (IQR)**
Male (n = 75)	531.4 (998.3)		1383.0 (2757.8)		2.0 (2.2)
Female (n = 60)	517.6 (924.7)	0.81	1210.6 (1791.0)	0.23	2.2 (2.7)
Age < 75 years n = 73 (54%)	669.6 (1042.0)		1608.9 (2508.6)		2.2 (2.2)
Age 75 years or over n = 62 (46%)	351.8 (954.0)	0.01	926.0 (2156.9)	<0.01	2.1 (2.5)
No stoma n = 76 (56%)	414.2 (903.30)		664.9 (1074.1)		2.2 (2.6)
Temporary stoma n = 18 (13%)	768.6 (1067.0)		1747.7 (1253.7)		2.1 (2.3)
Permanent stoma n = 40 (30%)	540.4 (960.3)	0.17	3766.3 (3381.2)	<0.05	1.6 (2.2)
Curative procedure n = 112 (83%)	456.5 (925.2)		1115.6 (2288.1)		2.5 (2.5)
Palliative procedure n = 23 (17%)	675.4 (1307.2)	<0.01	1815.2 (1806.0)	0.02	0.9 (1.3)
No adjuvant therapy n = 74 (56%)	394.8 (858.5)		1067.7 (2142.2)		1.3 (2.2)
Adjuvant therapy given n = 60 (44%)	633.6 (1158.1)	0.01	1739.6 (2478.3)	0.03	2.5 (2.0)
Stage of disease					
Dukes A n = 9 (7%)	61.0 (516.2)		1038.3 (5090.6)		3.1 (2.1)
Dukes B n = 34 (25%)	376.8 (1025.2)		1067.7 (2271.6)		2.1 (2.9)
Dukes C n = 73 (54%)	503.8 (860.8)		1141.3 (1948.1)		2.4 (2.3)
Dukes D n = 19(14%)	936.2 (1196.2)	<0.01	1815.2 (2092.5)	0.13	0.8 (0.9)
		<0.01**		0.06**	

* 2002 costs in pounds sterling, with qualification costs, discounted at 5% level.

** Cuzick test for trend.

**Table 3:** Univariate and Multivariate analysis by GP and total costs.

**Dependent variable**	**Ratio of geometric mean (95% confidence interval)**	
	**Univariate**	Multivariate**
**GP costs**		
Dukes A*	1	1
Dukes B	3.7 (1.5–8.8)	3.7 (1.6–8.7)
Dukes C	5.4 (2.4–12.2)	4.8 (2.1–10.7)
Dukes D	10.2 (4.0–26.0)	10.0 (4.0–25.0)
Adjuvant therapy		
No*	1	1
Yes	1.8 (1.2–2.8)	1.7 (1.1–2.6)
**Total costs**		
Dukes A*	1	1
Dukes B	1.4 (0.5–3.8)	Omitted by model
Dukes C	2.0 (0.8–5.3)	1.5 (2.0–6.0)
Dukes D	3.6 (1.2–10.9)	2.2 (1.2–3.8)
Stoma formed		
No*	1	1
Temporary	3.9 (2.2–6.7)	3.5 (2.0–6.0)
Permanent	7.7 (5.1–11.6)	7.4 (5.0–11.0)
Adjuvant therapy		
No*	1	1
Yes	1.7 (1.1–2.8)	1.5 (1.0–2.1)

* Baseline category.

** Backward stepwise linear regression.

## Abbreviations

GP: General Practitioner,
Dukes: Dukes stage of disease;
NHS: National Health Service.
